# Occurrence of virulence factors and carbapenemase genes in *Salmonella enterica* serovar Enteritidis isolated from chicken meat and egg samples in Iraq

**DOI:** 10.1186/s12866-022-02696-7

**Published:** 2022-11-22

**Authors:** Manal Hadi Ghaffoori Kanaan, Zena Kassem Khalil, Hawazin Thamir Khashan, Abdolmajid Ghasemian

**Affiliations:** 1 Department of Agriculture, Technical Institute of Suwaria, Middle Technical University, Baghdad, Iraq; 2 Optometry Department, Medical Technical Institute Al-Mansor, Middle Technical University, Baghdad, Iraq; 3 Department of Veterinary Public Health, Food Hygiene, College of Veterinary Medicine, University of Baghdad, Baghdad, Iraq; 4 Noncommunicable Diseases Research Center, Fasa University of Medical Sciences, Fasa, Iran

**Keywords:** Virulence factors, Carbapenemases, *Salmonella* Enteritidis, Iraq

## Abstract

**Background:**

Food-borne infections mainly due to *Salmonella enterica* serovar Enteritidis (*S*. Enteritidis) are major concerns worldwide. *S*. Enteritidis isolates may serve as reservoirs for spreading antimicrobial drug resistance genes including carbapenemases. This study aimed to screen the occurrence of virulence factors, carbapenemases, and antibiotic resistance genes in *S*. Enteritidis isolated from chicken meat and eggs in Iraq.

**Results:**

In total, 1000 non-duplicated chicken meat and 1000 egg samples were collected during 2019–2020. Presumptive *S*. Enteritidis isolates were initially identified by standard bacteriology tests and then were confirmed using polymerase chain reaction (PCR). Carbapenem resistance was detected using the disk diffusion method. Virulence and carbapenemase genes were screened using the PCR method. In total, 100 (5.0%) *S.* Enteritidi*s* isolates were identified from 2000 samples collected using phenotypic and molecular methods. These isolates were identified from 4.9% chicken meat (*n* = 49/1000) and 5.1% egg (*n* = 51/1000) samples, respectively. The most and the least susceptibility was found to gentamicin and ceftazidime antibiotics, respectively. The prevalence of different virulence factors were as follows: *phoP/Q* (40.0%), *traT* (30.0%), *stn* (22.0%), *slyA* (11.0%), and *sopB* (9.0%). Among 20 carbapenem-resistant *S.* Enteritidi*s* isolates, the most predominant carbapenemase gene was *bla*_IMP_ (35.0%, *n* = 7), followed by *bla*_OXA−48−like_ (25.0%, *n* = 5), and *bla*_NDM_ (10.0%, *n* = 2), while the *bla*_KPC_ and *bla*_VIM_ genes were not detected. The coexistence of *bla*_IMP_, *bla*_OXA−48−like_, and *bla*_NDM_ genes was determined in two isolates. The prevalence of different antibiotic resistance genes were as follows: *tetA* (87.1%), *tetB* (87.1%), *dfrA1* (77.6%), and *sul1* (83.6%).

**Conclusion:**

Considering the existence of carbapenem-resistant *S.* Enteritidi*s* harboring different virulence and antibiotic resistance genes in chicken meat and egg samples, adherence to proper hygienic conditions should be considered.

## Introduction

Spread of human pathogens including *Salmonella* strains which carry various virulence and antibiotic resistance genes is a serious concern in Iraq [1, 2]. *Salmonella* species cause a large part of food-poisoning cases and with more than 2500 serovars rank the most frequent causative agents of food poisoning in both developed and developing countries [3]. Some serovars of *Salmonella* are pathogenic for humans [4].

There are currently two species of *Salmonella*, *S. bongori* and *S. enterica* [5]. *Salmonella enterica* subspecies *enterica* serovar Enteritidis (*S*. Enteritidis) is a major causative agent of food-poisoning and gastroenteritis following consumption of meat, egg and milk with prosecutions of fever, abdominal cramps and diarrhea [5, 6]. There are various genes that associated with virulence of *Salmonella* located on either the chromosomes or the plasmid [6]. A number of factors contributed to *Salmonella* pathogenicity, including capsules, adhesions, flagella, and type 3 secretion systems (T3SSs) encoded on *Salmonella* pathogenicity islands (SPIs) 1–5 [5, 7]. Some of the virulence genes include *phoP/Q*, *stn*, *slyA*, *sopB*, *spvC*, and *traT* [5, 7, 8]. The interaction of the bacteria with the host may be affected by the expression of these factors, which ultimately determine the course of the infection [7].

Over the past few years, the rates of multidrug-resistant (MDR) *Salmonella* strains have increased across the globe [2, 9]. Additionally, several studies have examined the circulation of resistance genes within *Salmonella* strains at the molecular level [9]. However, one of the challenges that is now being addressed worldwide and rarely studied in *Salmonella* strains is the prevalence of carbapenemase genes that cause resistance towards carbapenem antibiotics. Infections caused by carbapenem-resistant bacteria are serious public health concerns because of the lack of treatment options for these infections. While carbapenemase-producing bacteria are documented in clinical infections, carbapenemase positive *Salmonella* strains, especially those that have food origins, are reported on a sporadic basis [10]. Increasing carbapenem resistance among foodborne pathogens necessitates active surveillance for these bacteria in the food chain, since they may be transmitted both to humans and the environment. *S*. Enteritidis isolates may serve as reservoirs and vehicles for spreading of carbapenemase genes. *Klebsiella pneumoniae* carbapenemase (KPC), New Delhi metallo-beta-lactamases (NDM), Verona integron-encoded metallo-b-lactamases (VIM), imipenem-carbapenemase (IMP), and carbapenem-hydrolysing oxacillinase (OXA) are among the carbapenemases that have been reported from food origin so far [11].

In Iraq, the occurrence of virulence factors and drug resistance genes in *S*. Enteritidis from food samples has rarely been studied. Hence, this study aimed to study the incidence of virulence factors and drug resistance genes in *S*. Enteritidis from food samples with focus on carbapenemase genes.

## Materials and methods

### Ethics

The present study was approved by the Technical Institute of Suwaria, Middle Technical University, Baghdad, Iraq. No human or live animal samples were examined in this study. All methods were carried out in accordance with relevant guidelines and regulations.

### Culture conditions and bacterial identification

This study was performed during an 8 month period from January to August 2018. In total, 2000 samples (1000 from fresh chicken meat and 1000 from eggs) were obtained from various chicken-producing centers (company products and traditional markets) in Baghdad, Iraq and rapidly transported to the laboratory for bacterial isolation. For chicken meat samples, 25 g was taken and homogenized and cultured into 225 mL trypticase soy broth containing 6.0% yeast extract (TSBYE) (Merck, Darmstadt, Germany) and incubated at 37 °C for overnight. Next, 1 mL of the cultured sample in the TSBYE was added into 9 mL of tetrathionate broth and incubated again. Then, 20 µL of this broth was cultured onto the xylose lysine deoxycholate (XLD) agar (Merck, Darmstadt, Germany) and CHROMagar™ Salmonella (Becton Dickinson GmbH, Germany) for bacterial isolates. The shell of the egg samples was disinfected with alcohol 75.0%. After the removal of the egg shells, the yolks and whites were mixed, and 25 g of the mixed sample was processed same as the meat sample. The colonies that appeared as reddish with/without black center on XLD or light mauve to mauve on CHROMagar™ Salmonella were picked for further confirmation using the API 20E test strips (BioMerieux, France). The isolates that identified by API 20E were screened for the presence of *sdfI* gene (*S*. Enteritidis serovar specific gene) using the polymerase chain reaction (PCR) with previously described primers (Table 1) [12]. The PCR was performed in a thermocycler (Applied Biosystems, Thermo Fisher Scientific, USA) with following program: initial denaturation (95 °C for 10 min), 30 cycles of: denaturation (94 °C for 1 min), annealing (60 °C for 90 s), and extension (72 °C for 90 s), and final extension at 72 ˚C for 5 min. The PCR was performed in a final volume of 25 µL containing following items: 4.0 µL 10X PCR buffer, 1.5 µL MgCl_2_, 1.5 µL dNTP (Fermentas, USA), 0.6 µL of each primers F and R, 5 µL Taq DNA polymerase (Fermentas), 1 µL DNA template, and 10.8 µL ddH_2_O. *S.* Enteritidis reference strain ATCC BAA-1587D-5™ was used as the positive control.

**Table 1:** Primers used for polymerase chain reaction (PCR) of this study

Genes	Primer sequence (5–3)	Annealing (°C)	Amplicon size (bp)	Reference
*slyA*	F: GCCAAAACTGAAGCTACAGGTG R: CGGCAGGTCAGCGTGTCGTGC	60	700	[8]
*phoP/Q*	F: ATGCAAAGCCCGACCATGACG R: GTATCGACCACCACGATGGTT	60	299	[8]
*sopB*	F: GATGTGATTAATGAAGAAATGCC R: GCAAACCATAAAAACTACACTCA	60	1170	[8]
*stn*	F: TTAGGTTGATGCTTATGATGGACACCC R: CGTGATGAATAAAGATACTCATAGG	60	617	[8]
*spvC*	F: ACTCCTTGCACAACCAAATGCGGA R: TGTCTTCTGCATTTCGCCACCATCA	60	129	[8]
*sdfI*	F: TGTGTTTTATCTGATGCAAGAGG R: TGAACTACGTTCGTTCTTCTGG	60	304	[12]
*bla*_IMP_	F: GGGTGGGGCGTTGTTCCTA R: TCTATTCCGCCCGTGCTGTC	62	198	[13]
*bla*_OXA−48−like_	F: CGCCCGCGTCGACGTTCAAGAT R: TCGGCCAGCAGCGGATAGGACAC	65	484	[13]
*bla*_NDM_	F: CGCACCTCATGTTTGAATTCGCC R: GTCGCAAAGCCCAGCTTCGC	61	1015	[13]
*bla*_KPC_	F: TTGCCGGTCGTGTTTCCCTTTAGC R: GGCCGCCGTGCAATACAGTGATA	64	282	[13]
*bla*_VIM_	F: CATTGTCCGTGATGGTGATGAGT R: GCGTGTCGACGGTGATGC	61	205	[13]
*traT*	F: GGTGTGGTGCGATGAGCACAG R: CACGGTTCAGCCATCCCTGAG	63	290	[14]
*sul1*	F: TTCGGCATTCTGAATCTCAC R: ATGATCTAACCCTCGGTCTC	57	822	[14]
*dfrA1*	F: GGAGTGCCAAAGGTGAACAGC R: GAGGCGAAGTCTTGGGTAAAAAC	55	367	[14]
*tetA*	F: GGTTCACTCGAACGACGTCA R: CTGTCCGACAAGTTGCATGA	55	577	[14]
*tetB*	F: CCTCAGCTTCTCAACGCGTG R: GCACCTTGCTGATGACTCTT	56	634	[14]

### Antibiotic resistance patterns

The antibiotic resistance profile of *S.* Enteritidi*s* isolates was performed on Mueller Hinton agar (MHA) (Merck, Darmstadt, Germany) using the disk diffusion method according to the Clinical and Laboratory Standards Institute (CLSI) [15]. The antibiotics ampicillin (10 µg), trimethoprim-sulfamethoxazole (1.25/23.75 µg), cefoxitin (30 µg), ceftazidime (30 µg), tetracycline (30 µg), imipenem (10 µg), meropenem (10 µg), and gentamicin (10 µg) (MAST, UK) were used. *Escherichia coli* ATCC 25,922™ was used for quality control. Isolates that were resistant to imipenem, meropenem or both antibiotics were considered carbapenem-resistant and selected for the detection of the carbapenemase genes using PCR.

### PCR screening of the virulence factors, carbapenemase, and some drug resistance genes

**Fig. 1:**
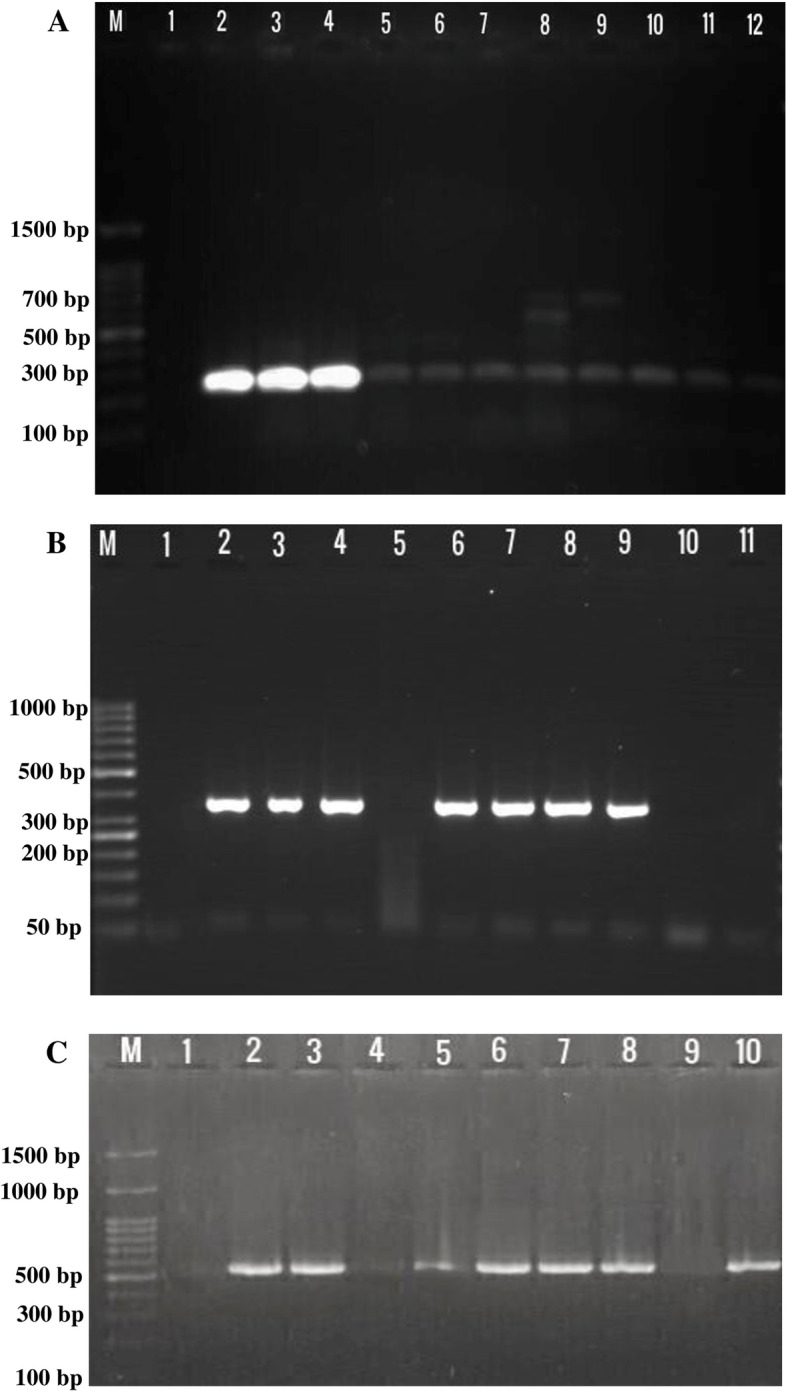
**A** Multiplex-PCR for virulence genes. M: DNA ladder (100 bp); Lane 1: control negative: DNA/RNA free water; Lanes 2–7 and 10–12: isolates positive for *phoP/Q* gene (299 bp); Lane 8: isolate positive for *phoP/Q* (299 bp), *stn* (617 bp), and *slyA* (700 bp); Lane 9: isolate positive for *phoP/Q* (299 bp) and *slyA* (700 bp). **B** Simplex PCR for *dfrA1* (367 bp) gene. M: DNA ladder (50 bp); Lane 1: control negative: DNA/RNA free water; Lanes 2: control positive; Lanes 2–4 and 6–9: isolates positive for *dfrA1* (367 bp) gene; Lanes 5, 10, and 11: isolates negative for *dfrA1* gene. **C** Simplex PCR for *tetA* (577 bp) gene. M: DNA ladder (100 bp); Lane 1: control negative: DNA/RNA free water; Lane 2: control positive; Lanes 3 and 5–8: isolate positive for *tetA* (577 bp) gene; Lines 4 and 9: isolates negative for *tetA* gene

For DNA extraction, the isolates were cultured onto the trypticase soy broth (TSB) (Merck, Darmstadt, Germany) medium for an overnight. Next, the AccuPrep Genomic DNA Extraction Kit (Bioneer, South Korea) was used for total genomic extraction according to the manufacturer’s guidelines. Then, a multiplex-PCR (M-PCR) was performed for the detection of the *phoP/Q, stn, slyA, sopB*, and *spvC* genes using prior described primers (Table 1) (Fig. 1A) [8]. The M-PCR was performed in a final volume of 25 µL with similar conditions of *sdfI* gene. Although the amount of ddH_2_O added was 4.8 µL. To screen the presence of the *traT* gene, carbapenemase genes (*bla*_IMP_, *bla*_OXA−48−like_, *bla*_NDM_, *bla*_KPC_ and *bla*_VIM_), and some other drug resistance genes including *sul1* and *dfrA1* (for trimethoprim-sulfamethoxazole), and *tetA* and *tetB* (for tetracycline), conventional PCR was performed with similar conditions of *sdfI* gene using specific primers (Table 1) (Fig. 1B and C) [13, 14]. Positive control genes were prepared from previous strains harboring studied genes that were kept in our laboratory. DNA/RNA free water was used as control negative in each PCR run.

#### Data analysis

The data was analyzed and presented as descriptive statistics using the statistical package for social science (SPSS) version 20.0 (IBM Corporation, Armonk, NY, USA). The chi^2^ test (or Fisher’s exact test if the numbers were small) was used to analyze the possible correlation between variables. If the *P*-value was smaller than the 0.05, the correlation was considered statistically significant [16, 17].

## Results

### Bacterial isolates

In total, 100 (5.0%) presumptive *S. enterica* isolates were identified from 2000 samples collected using phenotypic and API methods. These isolates were identified from 4.9% chicken meat (*n* = 49/1000) and 5.1% egg (*n* = 51/1000) samples, respectively. All isolates showed the 304 bp *sdfI* gene band in PCR and were confirmed as *S.* Enteritidi*s*. There was no significant difference between the prevalence of *S.* Enteritidi*s* in chicken meat samples with its occurrence in egg samples (*P*-value = 0.918).

### Antibiotic resistance patterns

The resistance rates of 100 *S.* Enteritidi*s* isolates were as follows: ampicillin (65.0%, *n* = 65), trimethoprim-sulfamethoxazole (67.0%, *n* = 67), cefoxitin (68.0%, *n* = 68), ceftazidime (78.0%, *n* = 78), tetracycline (70.0%, *n* = 70), imipenem (20.0%, *n* = 20), meropenem (18.0%, *n* = 18), and gentamicin (0.0%, *n* = 0). The most and the least susceptibility was found to gentamicin and ceftazidime antibiotics, respectively. The majority of isolates (65.0%, n = 65) were simultaneously resistant to ampicillin, trimethoprim-sulfamethoxazole, cefoxitin, ceftazidime, and tetracycline and were considered as MDR *S.* Enteritidi*s*. In total, 20.0% (*n* = 20) of isolates were carbapenem-resistant of which 18 isolates were simultaneously resistant to both imipenem and merpenem. These 20 isolates were further investigated for the presence of *bla*_IMP_, *bla*_OXA−48−like_, *bla*_NDM_, *bla*_KPC_ and *bla*_VIM_ genes.

### Prevalence of virulence factors

The prevalence of different virulence factors were as follows: *phoP/Q* (40.0%), *traT* (30.0%), *stn* (22.0%), *slyA* (11.0%), and *sopB* (9.0%) (Table 2). The *phoP/Q* was the most prevalent virulence factor, while the *spvC* was not detected. There were no significant difference between the occurrences of virulence genes in isolates that were collected from chicken meat with those of egg samples (Table 2).

**Table 2:** The frequency rates of virulence factors among 100 *Salmonella* Enteritidis isolates in meat and egg samples

Virulence factor	Total (*n* = 100) n (%)	Meat *(n* = 49) n (%)	Egg (*n* = 51 ) n (%)	*P*-value n (%)
*slyA*	11 (11.0)	5 (10.2)	6 (11.8)	> 0.999
*traT*	30 (30.0)	15 (30.6)	15 (29.4)	> 0.999
*phoPQ*	40 (40.0)	23 (46.9)	17 (33.3)	0.221
*sopB*	9 (9.0)	4 (8.2)	5 (9.8)	> 0.999
*sty*	22 (22.0)	10 (20.4)	12 (23.5)	0.811

### Prevalence of carbapenemase genes

Among 20 carbapenem-resistant *S.* Enteritidi*s* isolates, the most predominant carbapenemase gene was *bla*_IMP_ (35.0%, *n* = 7), followed by *bla*_OXA−48−like_ (25.0%, *n* = 5), and *bla*_NDM_ (10.0%, *n* = 2), while the *bla*_KPC_ and *bla*_VIM_ genes were not detected. The *bla*_IMP_ gene was detected in four meat and three egg samples, *bla*_OXA−48−like_ was found in three chicken meat and two egg samples, and *bla*_NDM_ was existed only in two meat samples. The co-existence of *bla*_IMP_, *bla*_OXA−48−like_, and the *bla*_NDM_ genes was found in 2 (10.0%) isolates with meat origin.

### Prevalence of other drug resistance genes

Among 70 tetracycline-resistant *S.* Enteritidi*s* isolates, the prevalence of resistance genes was as follows: *tetA* (87.1%, *n* = 61) and *tetB* (87.1%, *n* = 61). Also, in the 67 trimethoprim-sulfamethoxazole-resistant isolates, the prevalence of resistance genes was as follows: *dfrA1* (77.6%, *n* = 52) and *sul1* (83.6%, *n* = 56).

## Discussion

Zoonotic pathogens including *Salmonella* serovars are commonly transmitted by chickens and eggs [18]. *S.* Enteritidi*s* is an important *Salmonella* serovar which causes zoonotic infections. In this study, the total prevalence rate of *S.* Enteritidi*s* in chicken meat and egg samples was 5.0%. The *S.* Enteritidi*s* strains were isolated from 49 (4.9%) chicken meat and 51 (5.1%) eggs samples, respectively. In consistent with the current research, Xie et al. [19] from China, reported a very similar prevalence of *Salmonella* serovars (5.4%, *n* = 54/1000) in egg samples. Also, our findings were in line with the World Health Organization (WHO) data about Asian countries [20]. According to the WHO global foodborne infections network data, the prevalence of *S.* Enteritidis serovar contamination in poultry samples ranged from 5 to 93.7% in Asia and Europe and from 19.2 to 49% in Africa [20]. According to a report by Pijnacker et al. [21], between May 2015 and Oct 2018, 1209 outbreak cases of *S.* Enteritidis linked to food products including eggs were identified in 18 European Union (EU) and the European Economic Area (EEA) countries. In another study by Almashhadany [22] from Iraq, 7.1% (*n* = 16/225) of grilled chicken meat samples were positive for *Salmonella* species that was slightly higher than the current study. He reported that 12.5% (*n* = 2/16) of all isolated *Salmonella* species were *S.* Enteritidis [22]. In a study by Bahramianfard et al. [6] from Iran, 1.3% of egg samples were contaminated with *S*. Enteritidis that was lower than our observation. In Tunisia, a prevalence rate of 16.0% has been reported for *S*. Enteritidis in chicken carcasses [23]. Various countries have different hygienic control and management programs, which may explain the differences in *S.* Enteritidis contamination rates of food products [6]. The *Salmonella* species in infected poultry feces may contaminate the eggshells and penetrate the interior of eggs, causing bacteria to grow inside [18]. Another reason for these differences may be due to the detection method, sample size, and sample types.

In this study, more than 50.0% of *S.* Enteritidis isolates were simultaneously resistant against ampicillin, trimethoprim-sulfamethoxazole, cefoxitin, ceftazidime, and tetracycline and considered as MDR isolates. This MDR rate was higher than a previous report from Iran (27.0%) [6], but lower than reports from Egypt (75.7% and 100.0%) [18, 24]. However, in this study, imipenem, meropenem, and gentamicin were the most effective antibiotics. In a previous study from Iran, a lower resistance rates had been reported for ceftazidime (11.1%) and trimethoprim-sulfamethoxazole (20.6%) [6]. In this study, the high resistance rate against third-generation cephalosporins such as ceftazidime may be explained by the presence of extended-spectrum beta-lactamase (ESBL) enzymes which were not investigated [24, 25]. In recent years, ESBLs have spread among *Salmonella* species in different countries [24, 25]. The lower resistance rates against carbapenems including imipenem and meropenem in comparison with other antibiotic classes may be as a result of limited use of these antimicrobials in poultry farms that was consistent with previous reports from Bangladesh [9, 26] and Egypt [18].

Also, contrary to the current study, Gritli et al. [23] from Tunisia, reported a lower resistance rates against tetracycline (13.0%) and trimethoprim-sulfamethoxazole (0.0%). However, they reported the gentamicin as one of the most effective antibiotics that was in parallel with our observations. The most common antibiotic used in poultry has historically been tetracyclines. Therefore, resistance can be occurred in a variety of ways in bacteria, including changing ribosomal targets, efflux pumps, and inactivating enzymes [23]. In this study, the presence of *tetA* (87.1%)/*tetB* (87.1%) and *dfrA1* (77.6%)/*sul1* (83.6%) genes may be contributed to the high resistance rates against tetracycline and trimethoprim-sulfamethoxazole, respectively. In line with our findings, in a previous study by Abou Elez et al. [18] from Egypt, high resistance rates were observed against ampicillin (100.0%) and tetracycline (88.0%). They stated that the presence of the *tet* genes may be responsible for this high resistance rate to tetracycline. In their study, the most *S.* Enteritidis isolates (84.0%, *n* = 21/25) harbored *tetA* gene, whereas the *tetB* gene was only found in 2 isolates [18]. However, in another report by Shittu et al. [27] from Nigeria, the *tetA* and *tetB* genes were not detected in any *S. enterica* serovars including *S.* Enteritidis isolates that was in contrast to our results. In another research by Siddiky et al. [26] from Bangladesh, *tetA* and *sul1* genes were detected in 3.44% of *S.* Enteritidis isolates that was lower than our study. Arkali and Çetinkaya from Turkey [28] detected the *sul1* and *tetA* with 57.8% and 34.4% proportions, respectively. Also, El-Tayeb et al. [29] from Saudi Arabia stated that *tetA* and *dfrA1* were the most prevalent genes accountable for resistance to tetracycline and trimethoprim-sulfamethoxazole, respectively. Discrepancies among studies may be due to the difference in sample type, sample size, and antibiotic use pattern in different regions.

As a result of the presence of virulence factors and antibiotic resistance genes, microbes can become more pathogenic [9]. In this study, the *phoP/Q* gene was the predominant virulence gene (40%), followed by *traT, stn, slyA* and *sopB* with frequencies of 30%, 22%, 11% and 9%, respectively. However, the *spvC* gene was not detected in any isolate. The absence of *spvC* gene, which is encoded by a plasmid, is possibly due to the loss of plasmid among *S.* Enteritidis isolates. Contrary to the current study, in a previous report by Hai et al. [30] from China, more than 90.0% of the *Salmonella* isolates harbored *stn* and *sopB* genes. Also, Wang et al. [31] from China reported higher incidence rates for *sopB* (100.0%) and *spvC* (93.8%) genes among *Salmonella* serovars isolated from 120 retail meat samples, including chicken (*n* = 45), duck (*n* = 30), and pork (*n* = 45). In the prior studies from Iran [6], Tunisia [23], and Nigeria [27], the prevalence rates of *spvC* gene were 50.8%, 45.8%, and 59.1%, respectively. In line with our results, Khodadadipour et al. [32] from Iran detected the *phoP/Q* gene (33.3%) as the most prevalent virulence determinant in *S*. Enteritidis strains isolated from meat and egg samples. Also, they stated that all isolates were negative for *spvC* gene [32]. Takaichi et al. [33] from Indonesia reported prevalence rates of 60.0%, 86.0%, and 88.0% for *slyA*, *phoP/Q*, and *sopB*, respectively. Also, they did not identify the *spvC* gene that was in consistent with the current research. The *stn* gene was found in 100.0% (*n* = 25) of *S.* Enteritidis isolates [18] and 40.0% (*n* = 48/120) of the *Salmonella* serovars [34] in Egypt that was higher than the current study. Although the prevalence rate of virulence factors examined in this study was lower than in other countries, it cannot be concluded that our strains are less pathogenic. Because *Salmonella* serovars possess several virulence factors that were not investigated here. There is evidence that the high frequency of detection of virulence factor genes in *Salmonella* isolates highlights their importance in human health [31].

There is still a very low incidence of carbapenem resistance in *Salmonella* serovars as compared to other *Enterobacteriaceae* species [35, 36]. However, 20.0% (*n* = 20/100) of *S*. Enteritidis isolates were carbapenem-resistant in the current study that was higher than the previous reports from Argentina (0.0%) [7], Bangladesh (0.0%) [9], and Poland (0.0%) [12]. The sale of antibiotics without a doctor prescription in most pharmacies, self-administration of antibiotics, their suboptimal use in the agricultural and food industries can be possible reasons for creating this selective pressure for the spread of carbapenem-resistant strains. Hence, the presence of 5 carbapenemase genes (*bla*_IMP_, *bla*_OXA−48−like_, *bla*_NDM_, *bla*_KPC_ and *bla*_VIM_) was investigated in these 20 carbapenem-resistant *S*. Enteritidis isolates. To the best of our knowledge, this was the first study in Iraq that shed light on the prevalence of carbapenemase genes in *S*. Enteritidis isolates from chicken meat and egg samples. The PCR assay revealed the *bla*_IMP_ (35.0%, *n* = 7) as the most predominant carbapenemase gene, followed by *bla*_OXA−48−like_ (25.0%, *n* = 5), and *bla*_NDM_ (10.0%, *n* = 2), while the *bla*_KPC_ and *bla*_VIM_ genes were not detected. The presence of carbapenemase genes in *S.* Enteritidis isolated from food products has been investigated in few studies [35]. So far, various occurrence rates of *bla*_IMP_, *bla*_KPC_, *bla*_NDM_, *bla*_OXA−48−like_, *bla*_SPM_, and *bla*_VIM_ carbapenemase genes in *Salmonella* strains of food origin have been reported [35, 37–39]. Abdel-Kader et al. [37] form Egypt, detected the *bla*_KPC_ gene in 5 of 13 *S. enterica* strains isolated from chicken giblets. However, *bla*_OXA−48_ and *bla*_NDM_ genes were not identified [37]. In another study from Egypt, 2 isolates from chicken meat contained the *bla*_OXA−48−like_ gene and two isolates harbored *bla*_NDM_ but none of the egg samples were infected by carbapenemase-positive *Salmonella* serovars [39]. Ghazaei [37] from Iran, reported the presence of *bla*_IMP_ (31.57%), *bla*_SPM−1_ (10.52%), and *bla*_VIM_ (57.8%) genes in *S. enterica* strains isolated from poultry meat. Contrary to our findings, Gawish et al. [24] did not find any carbapenemase gene in *S. enterica* isolates. According to the results obtained in this study, it seems that more extensive studies focusing on food products are needed around the world to provide a more accurate estimation of prevalence of carbapenemase genes in *Salmonella* strains with food origin.

### Limitations

The current study had some limitations due to lack of financial resources. This study focused only on poultry products and did not investigated other food products. The prevalence of ESBLs, AmpC, and other carbapenemases were not investigated. The carbapenemase genes were not sequenced. The clonal relatedness of carbapenem-resistant isolates was not evaluated. It is recommended to perform more in-depth studies in future to analyze the precise mechanisms contributed to the antibiotic resistance in bacterial isolates from food products in Iraq.

## Conclusion

The current study was the first in Iraq to evaluate the prevalence, antibiotic resistance patterns, virulence factors, and antibiotic resistance genes including carbapenemases of *S*. Enteritidis in the chicken meat and egg samples. A relatively high rate of MDR *S*. Enteritidis among the poultry products necessitate control of antibiotic usage in chicken farms in the studied region. Also, the emergence of virulent *S*. Enteritidis isolates carrying carbapenemase genes particularly *bla*_NDM_ with resistance to last-line antibiotic resorts is a concern toward control and eradication of these isolates. Our data provides insights regarding the attention about environmental sources, particularly poultry role in the antibiotic resistance spread. One health instructions should be considered for the safe production of *Salmonella*-free food products for consumers.

## Data Availability

All data generated or analyzed during this study are included here and are available from the corresponding author on reasonable request.

## Abbreviations

IMP: Imipenem-carbapenemase
KPC: *Klebsiella pneumoniae* carbapenemase
MDR: Multidrug-resistant
NDM: New Delhi metallo-beta-lactamases
OXA: oxacillinase
SPIs: *Salmonella* pathogenicity islands
T3SSs: Type 3 secretion systems
VIM: Verona integron-encoded metallo-b-lactamase
